# *pinkIndexer* – a universal indexer for pink-beam X-ray and electron diffraction snapshots

**DOI:** 10.1107/S2053273319015559

**Published:** 2020-01-10

**Authors:** Yaroslav Gevorkov, Anton Barty, Wolfgang Brehm, Thomas A. White, Aleksandra Tolstikova, Max O. Wiedorn, Alke Meents, Rolf-Rainer Grigat, Henry N. Chapman, Oleksandr Yefanov

**Affiliations:** a Center for Free-Electron Laser Science, Deutsches Elektronen-Synchrotron DESY, Notkestraße 85, 22607 Hamburg, Germany; b Vision Systems, Hamburg University of Technology, 21071 Hamburg, Germany; c Department of Physics, Universität Hamburg, Luruper Chaussee 149, 22761 Hamburg, Germany; d The Hamburg Center for Ultrafast Imaging, Universität Hamburg, Luruper Chaussee 149, 22761 Hamburg, Germany

**Keywords:** indexing, *pinkIndexer*, *CrystFEL*, pink X-ray beam, serial electron diffraction

## Abstract

A crystallographic indexing algorithm, *pinkIndexer*, is presented for the analysis of snapshot diffraction patterns. It can be used in a variety of contexts including measurements made with a monochromatic radiation source, a polychromatic source or with radiation of very short wavelength. As such, the algorithm is particularly suited to automated data processing for two emerging measurement techniques for macromolecular structure determination: serial pink-beam X-ray crystallography and serial electron crystallography, which until now lacked reliable programs for analyzing many individual diffraction patterns from crystals of uncorrelated orientation. The algorithm requires approximate knowledge of the unit-cell parameters of the crystal, but not the wavelengths associated with each Bragg spot. The use of *pinkIndexer* is demonstrated by obtaining 1005 lattices from a published pink-beam serial crystallography data set that had previously yielded 140 indexed lattices. Additionally, in tests on experimental serial crystallography diffraction data recorded with quasi-monochromatic X-rays and with electrons the algorithm indexed more patterns than other programs tested.

## Abstract 2

*pinkIndexer*, an algorithm developed for indexing of snapshot diffraction patterns recorded with pink-beam X-rays, monochromatic X-rays and electrons, is described and its use evaluated.

## 1. Introduction   

Protein crystallography is a vibrant and continually evolving field spurred by the development of new radiation sources, detectors, measurement techniques and analysis methods. One example is the relatively recent development of serial crystallography using femtosecond-duration X-ray pulses from free-electron lasers (SFX), which is suited to the study of micron-sized and smaller macromolecular crystals (Chapman *et al.*, 2011 ▸; Boutet *et al.*, 2012 ▸; Schlichting, 2015 ▸; Gati *et al.*, 2017 ▸). With pulses that out-run atomic motions initiated by photoabsorption, doses may far exceed conventional radiation damage limits to provide structures of radiation-sensitive proteins free of obvious radiation damage, permitting time-resolved studies of biomolecular dynamics at physiologically relevant temperatures (Suga *et al.*, 2014 ▸; Tenboer *et al.*, 2014 ▸; Kang *et al.*, 2015 ▸; Pande *et al.*, 2016 ▸; Stagno *et al.*, 2016 ▸). The approach of measuring only a single snapshot diffraction pattern from each of many crystals also allows for a lower overall exposure per crystal, and hence lower doses than would be accrued in conventional rotation measurements. This, and the potential for high-throughput measurements, has motivated the development of serial crystallography at synchrotron radiation facilities (Stellato *et al.*, 2014 ▸; Nogly *et al.*, 2015 ▸) and using electron microscopes (Smeets *et al.*, 2018 ▸; Bücker *et al.*, 2019 ▸).

The speed of serial crystallography measurements, and often the corresponding consumption of sample, is primarily limited by the radiation fluence on the sample and the detector frame rate. At synchrotron radiation sources, higher fluences can be obtained by foregoing the monochromator and using a polychromatic beam. Combined with the enhanced coverage of reciprocal space, a moderate bandwidth of the order of a few per cent (referred to as a ‘pink’ beam) may offer the additional advantage of fewer necessary diffraction patterns for a serial crystallography measurement (White *et al.*, 2013 ▸; Dejoie *et al.*, 2013 ▸). For example, Meents *et al.* (2017 ▸) demonstrated room-temperature serial crystallography with 100 ps exposure times using the full spectrum of an undulator harmonic (5% relative bandwidth), with the ability to determine structures from as little as 50 indexed diffraction patterns. However, the automated analysis of pink-beam diffraction patterns has been found to be problematic, with only 15% of patterns successfully indexed in the demonstration of Meents *et al.* (2017 ▸). We were therefore motivated to create a new robust algorithm to index snapshot diffraction patterns recorded with a quasi-collimated beam of arbitrary bandwidth, with the requirement to index weak or incomplete patterns, using approximately known unit-cell parameters. In meeting this goal, we produced an algorithm ‘*pinkIndexer*’. We found that *pinkIndexer* can also be applied to several other data collection methods. In addition to superior performance in processing pink-beam diffraction compared with the state-of-the-art algorithms, the algorithm indexes more patterns in monochromatic serial crystallography data sets than all other programs tested, and is successful in indexing snapshot crystal diffraction patterns recorded with electrons.

The determination of the 3D macromolecular crystal structure requires the measurement of diffraction intensities at reciprocal-lattice points throughout a volume of reciprocal space, yet a single snapshot diffraction pattern accesses just a cut through this space. Conservation of photon energy and momentum dictates that for a specific X-ray wavelength this cut is given by a spherical surface – the Ewald sphere – which passes through the origin of reciprocal space. In a serial diffraction experiment the reciprocal-space volume (reduced by the symmetry of the crystal) is sampled by many such patterns recorded from crystals at various random orientations. Usually the orientation of each crystal is initially unknown, and therefore so too is the orientation of its reciprocal lattice. A key analysis step is to identify the crystal orientation, which is equivalent to providing the correct indices to the observed diffraction spots. Furthermore, the distribution of crystal orientations is usually assumed to be random, precluding the use of correlations between successive patterns to deduce the crystal orientations. In the case of a broad-bandwidth X-ray beam, indexing is complicated by the uncertainty of the particular incident wavelength that gave rise to a given Bragg spot, while for electron diffraction the short wavelength results in an almost flat Ewald sphere for which the determination of unknown 3D lattice parameters is ill conditioned. Indeed, the main bottleneck in pink-beam and electron serial crystallography analysis has been the indexing step.

Automatic indexing algorithms implemented in widely used software including *MOSFLM* (Powell, 1999 ▸), *XDS* (Kabsch, 1993 ▸, 2010 ▸) and *DirAx* (Duisenberg, 1992 ▸) were originally devised for data collected in a rotation series with monochromatic radiation. They typically perform poorly when presented with individual pink-beam or electron snapshot diffraction patterns due to their reliance on the particular conditions of monochromatic rotation measurements. Recent algorithms designed for indexing snapshot diffraction patterns encountered in serial crystallography include *TakeTwo* (Ginn *et al.*, 2016 ▸), *FELIX* (Beyerlein *et al.*, 2017 ▸) and *XGANDALF* (Gevorkov *et al.*, 2019 ▸). These all assume monochromatic radiation and do not fare much better than other indexers when processing polychromatic diffraction patterns. Several indexing approaches have been developed for polychromatic crystal diffraction, also referred to as Laue diffraction (Moffat, 1997 ▸). These include an approach due to Jacobson (1986 ▸) that requires the use of an energy-resolving position-sensitive detector; the Daresbury software suite for indexing Laue patterns (Helliwell *et al.*, 1989 ▸; Campbell *et al.*, 1998 ▸) and the *Precognition* software (Ren *et al.*, 1999 ▸) based on searching arcs of reflections so that prominent zone axes can be identified; geometric approaches of Carr *et al.* (1993 ▸) and Wenk *et al.* (1997 ▸); and the *LaueUtil* toolkit (Kalinowski *et al.*, 2011 ▸) which carries out a clustering analysis of possible orientations that map lattice vectors to observed peaks. The latter algorithm requires measurements of a crystal at several known relative orientations and is therefore not suited to serial crystallography. Of these, the current state-of-the-art software for indexing single wide-bandwidth diffraction patterns of macromolecular crystals is *Precognition*. However, while this works well for patterns recorded with a very wide spectrum (*e.g.* that of a wiggler or bending magnet where the bandwidth is more than 10% of the nominal X-ray energy), it becomes less reliable as the number of Bragg spots decreases as occurs with either reduced spectral width (less than 5% of the nominal X-ray energy) or with small crystals, where only several tens of Bragg reflections are observed.

Here we present the principles and performance of our general indexing algorithm, *pinkIndexer*. As described in Section 2, the algorithm maps observed Bragg reflections into trajectories of possible lattice orientations. The most likely orientation is then determined as the orientation in which most trajectories intersect. As such, *pinkIndexer* covers the cases of monochromatic serial X-ray crystallography, X-ray crystallography using the unmodified spectrum of an undulator of 1% to 25% bandwidth and approximately 1 Å wavelength, and serial electron crystallography at approximately 0.01 Å wavelength. These cases are evaluated in Section 3. The algorithm can be employed in automated processing of serial crystallography data sets, for example using the *CrystFEL* sofware suite (White *et al.*, 2012 ▸; White, 2019 ▸).

## 2. The *pinkIndexer* algorithm   

### 2.1. Diffraction geometry   

**Figure 1:**
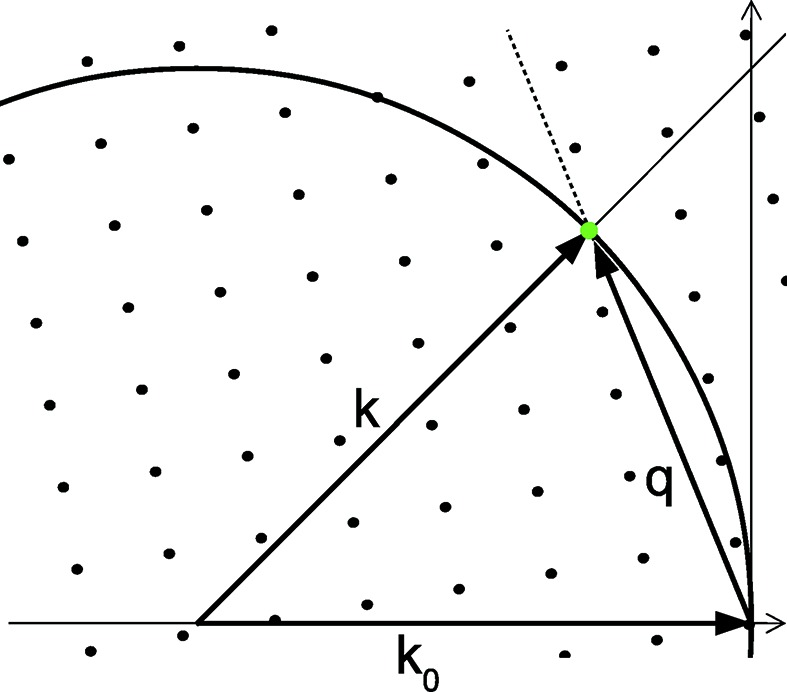
A 2D representation of reciprocal space, showing RLPs of a crystal and the Ewald sphere for monochromatic diffraction. 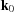 is the wavevector of the incident beam, 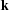 is the outgoing wavevector elastically scattered from the structure in the object with spatial frequency 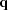. The diffraction intensity measured by a detector in the direction of vector 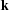 corresponds to the intensity at the green RLP.

Consider elastic scattering from an object by a plane monochromatic wave characterized by a wavevector 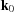 with a wavelength 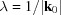. In the kinematic approximation, the strength of scattering in a direction 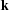 is given by the magnitude of the Fourier component of the object at a spatial frequency 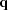 equal to the momentum transfer 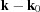 (James, 1950 ▸). Elastic scattering (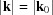) confines the observable spatial frequencies 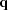 to the Ewald sphere, shown as a circle in Fig. 1 ▸. Each pixel in a detector placed in the far field measures a particular direction given by the unit vector 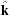, which unambiguously maps to a point 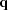 in reciprocal space. The spatial frequency spectrum of a crystal of infinite extent is a lattice of points that are commonly referred to as reciprocal-lattice points (RLPs), shown as black dots in Fig. 1 ▸. As can be seen in that figure, a diffraction spot observed in a particular direction 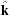 is unambiguously mapped to a particular RLP (green dot in Fig. 1 ▸).

**Figure 2:**
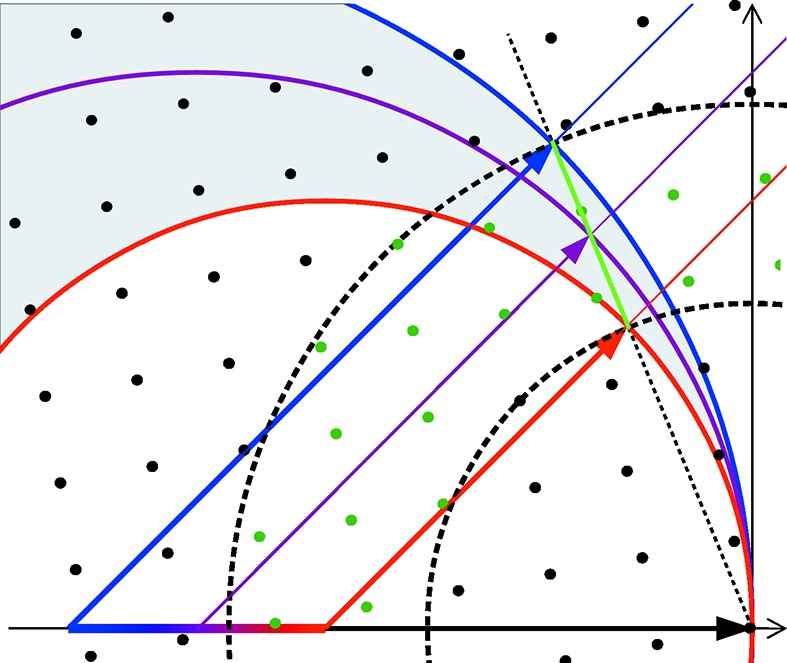
A 2D representation of reciprocal space depicting Ewald spheres for polychromatic diffraction spanning the longest wavelength shown in red to the shortest in blue. The volume between the red and blue spheres is excited and the intensity measured at the detector in the direction of the highlighted vectors corresponds to the integral of diffraction intensities along an uncertainty line segment (ULS) (green line). Candidate RLPs (dark green dots) lie in the volume between shells depicted by the dashed circles. Note that the red, purple and blue vectors are parallel and therefore the corresponding photons arrive at the same pixel of the detector. However, since the photons have different wavelengths, they do not interfere coherently and their intensities are simply added together.

Consider now the case where the radiation source emits a finite but continuous distribution of wavelengths within some known range. Instead of a single Ewald sphere as in the case of monochromatic illumination, each incident wavelength produces an Ewald sphere with a radius inversely proportional to that wavelength. Thus a volume of reciprocal space can be excited in a diffraction experiment, contributing to the 2D diffraction pattern. This volume is depicted in Fig. 2 ▸, bounded by the red and blue Ewald spheres (longest to shortest wavelengths in the range). There is a significant difference to the monochromatic case: with a polychromatic source, a particular scattering direction 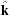 no longer maps to a single point in reciprocal space. There may be many diffracted wavevectors, each with a different wavelength (and hence different wavevector magnitude and different placement in the Ewald-sphere construction), but pointing in the same direction 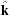 and thus arriving at the same point on the detector. These wavevectors are depicted by the red, purple and blue arrows in Fig. 2 ▸. Turning this around, for a given diffraction direction 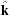, there are many points 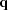 in reciprocal space that contribute to the diffracted intensity. All these points lie on a straight line segment (green line in Fig. 2 ▸), the extension of which passes through the origin of reciprocal space. The line segment can be described by 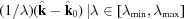.

We therefore see that, in the case of broad bandwidth, a point on the detector integrates signal from a line segment in reciprocal space, in contrast to a single point in the monochromatic case. The RLP which generates a Bragg peak observed at some position on the detector may therefore lie anywhere on the corresponding line segment. We call this line segment the uncertainty line segment (ULS), shown in green in Fig. 2 ▸. The main challenge for analyzing broad-bandwidth snapshot crystal diffraction patterns is to determine where along the ULS is the RLP which generated the observed Bragg peak. This is equivalent to identifying the wavelength that excited the measured RLP. Note that if more than one RLP lies on the ULS, they will contribute to the observed intensity, excited by different wavelengths. The bandwidth in that case would be too broad to distinguish those particular reflections in the peak-finding stage without energy-resolving detectors. It is nevertheless possible to separate the summed intensities after indexing and integration (Zurek *et al.*, 1985 ▸; Shrive *et al.*, 1990 ▸).

Since the crystal orientation is not known, and thus the orientation of the reciprocal lattice is also not known, candidate RLPs may lie anywhere in the volume between 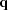 shells centered at the origin with radii set by the scattering direction and range of wavelengths as depicted by the dashed circles in Fig. 2 ▸. We call the RLPs that can match a ULS by rotation of the reciprocal lattice ‘candidate RLPs’ (candidates to predict the particular Bragg spot). The candidate RLPs are plotted in dark green in Fig. 2 ▸.

### 2.2. Determining the crystal orientation   

The task of indexing is to find the crystal orientation which gives rise to a particular measured diffraction pattern and then to assign indices to predicted reflection locations. In practice this is achieved by finding the crystal orientation which best predicts the set of Bragg peaks observed on the detector. We assume that the unit-cell parameters of the crystal are known. *pinkIndexer* determines the likely crystal orientation as follows. (i) For each Bragg spot observed on the detector, find all RLPs of a crystal that can be intersected by the Bragg spot’s ULS by rotation around the origin (candidate RLPs). (ii) For each observed Bragg spot, find all rotations of the crystal that place at least one candidate RLP onto the corresponding ULS. This is equivalent to finding all orientations of the reciprocal lattice that could predict the measured Bragg spot. (iii) Find the orientation which predicts the most Bragg spots from the list of candidate orientations for all Bragg peaks observed in the pattern. The orientation which correctly predicts the most observed Bragg spots will be the chosen indexing solution. (iv) Refine the lattice parameters and other experimental parameters to further improve the agreement of predicted and observed Bragg peaks [if the original parameters were not accurate, one could repeat steps (i) to (iv) using the refined parameters]. Once the crystal orientation is determined it is of course possible to predict the location and wavelength of all potential reflections including absent or weak reflections not present in the set of observed Bragg peaks. These can then be included in the integration of the observed intensities for structure determination.

**Figure 3:**
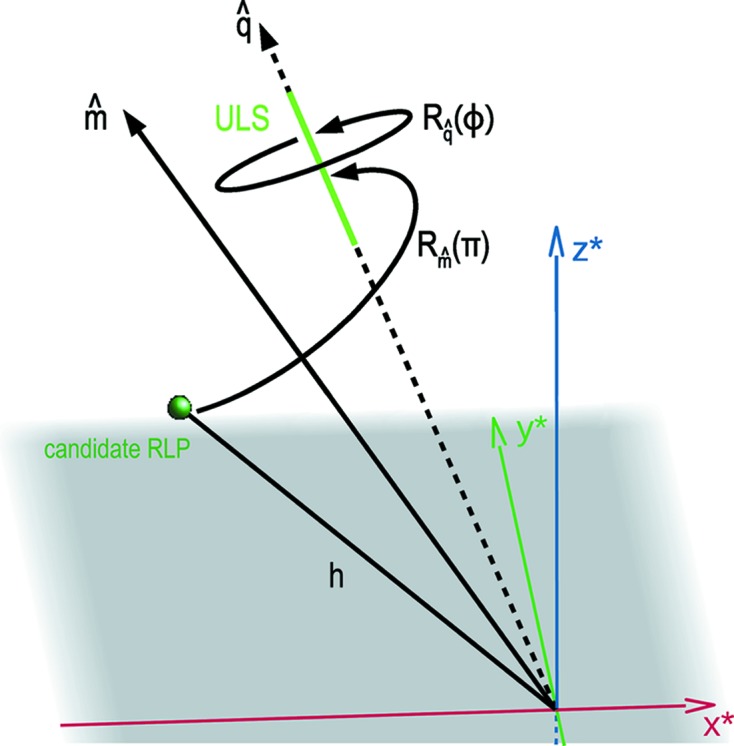
All possible rotations for which a candidate RLP intersects with a given ULS are uniquely described by the rotation 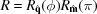. The first rotation 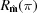 rotates the lattice by the constant angle of π around the bisecting vector 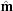 of 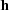 and 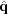. The second rotation 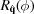 rotates the lattice around the axis 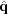 of the ULS by an angle ϕ.

The main challenge lies in making the search outlined above tractable and robust. As we will now discuss, for each candidate RLP 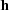 there is an infinite set of reciprocal-lattice rotations which place it onto its particular ULS. We identify all in this family of rotations by constructing a rotation operation in two steps: first the reciprocal lattice is rotated such that the vector 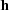 of the RLP is rotated by an angle π around the axis 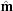 that bisects 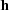 and 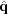 as shown in Fig. 3 ▸. This rotation brings the candidate RLP onto the ULS. Next, the reciprocal lattice is rotated around 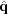 by a rotation of ϕ (see Fig. 3 ▸). Since the rotated candidate RLP lies on the ULS it is invariant to the second rotation and thus all rotations ϕ are potential orientations of the lattice. This construction is only valid for one particular candidate RLP and a particular ULS. The particular RLP might not actually give rise to the Bragg spot which generated the ULS. That is, none of the orientations of the lattice generated by the operations 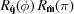 might be the indexing solution. In a triclinic lattice only one candidate RLP, as well as other RLPs lying on the same straight line through the origin, generate the correct indexing solution. In lattices with higher symmetry, multiple candidate RLPs generate correct indexing solutions.

**Figure 4:**
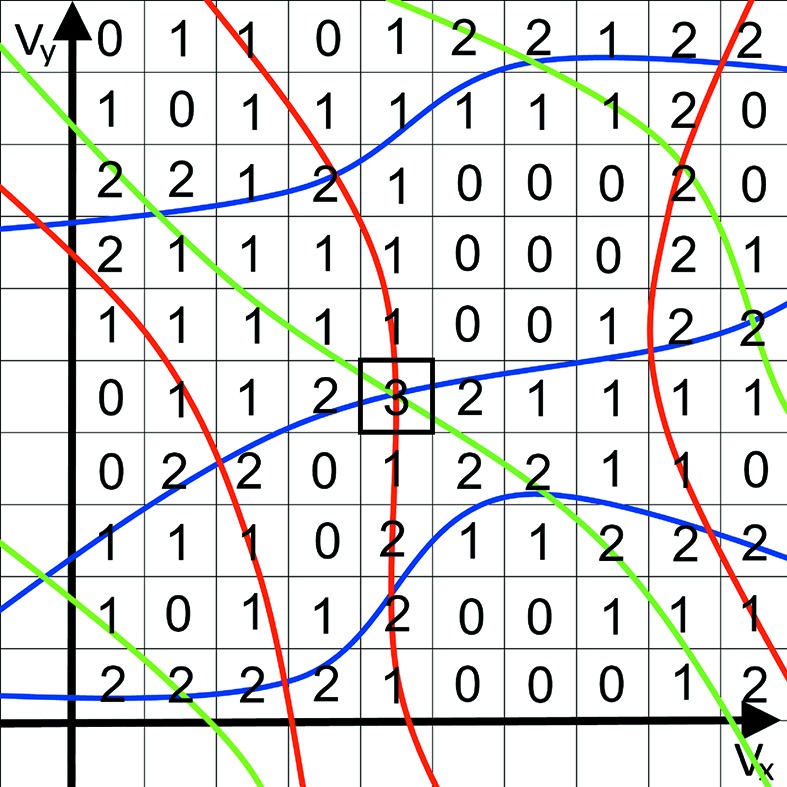
Schematic sketch of a rotogram in a 2D vector space. Each point in the rotogram corresponds to a rotation (defined by the constants 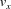 and 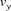) of a sample reciprocal lattice. Each color marks the positions in the rotogram that predict one particular Bragg spot. The position that is marked by most colors corresponds to the orientation that predicts most Bragg spots correctly – a good indexing solution.

To determine common orientations that bring a number of RLPs onto ULSs we define a 3D vector space that contains curves parameterized by 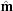, 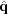 and the rotation angle ϕ, satisfying the reflection conditions stated above that place a particular candidate RLP onto a particular ULS (corresponding to an observed Bragg spot). The vector space consists of 3D since it is spanned by three variables describing rotations, such as the three Euler angles. In this 3D space, all candidate RLPs for a particular Bragg spot will form a set of non-intersecting curves. We call this collection a rotogram. By combining rotograms for all measured Bragg spots, a total rotogram for a diffraction pattern is formed, depicted schematically in Fig. 4 ▸. The point in a rotogram with the highest density of overlapping curves provides the lattice orientation that predicts most of the observed Bragg spots. This point represents the rotation of the lattice onto that of the measured crystal, *i.e.* it is the indexing solution. The task of crystal orientation determination is therefore now reduced to one of finding the point in rotation space with the largest number of intersecting lines.

### 2.3. Algorithm details   

In practice, many additional issues arise when dealing with data from a real experiment that complicate the indexing process, such as spurious intensity peaks resulting from experimental noise, or multiple crystals in the beam contributing to the same diffraction pattern. The robustness of the algorithm to these factors becomes a critical issue.

*pinkIndexer* uses the same basic approach as another indexing method for monochromatic crystal diffraction patterns, *FELIX* (Beyerlein *et al.*, 2017 ▸), which similarly parameterizes possible orientations as curves in a 3D rotation space. Both methods are similar to the Hough and Radon transforms that operate on 2D parameter spaces. With such approaches, the choice of the mapping function is crucial for the performance and simplicity of the algorithm. Well-known mappings for 3D rotations to a 3D space are: the Euler-angles representation, the axis-angle representation, the Gibbs representation and the modified Rodrigues parameters (Terzakis *et al.*, 2018 ▸). We employ a novel mapping function by which we achieve a drastic reduction of complexity and the number of necessary parameters compared with *FELIX* (which uses Rodrigues parameters), while at the same time increasing the noise tolerance. The following features of the transform are desired for robustness and efficient construction of the rotogram: (*a*) adjacent voxels in the rotogram correspond to similar rotations; (*b*) rotations are distributed uniformly across a volume of the 3D rotation space; (*c*) the results of the transformation are efficiently discretized in cuboid samples (*i.e.* on an orthogonal lattice); (*d*) the transform is calculable in an efficient way.

Since none of the well-known examples sufficiently fulfill these requirements, we propose another transform that better fulfills the requirements and is the major factor in the quality of the *pinkIndexer* algorithm. In our scheme, a single rotation operation 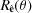 is determined from the composite rotation 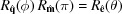. This rotation then is mapped to the point 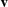 in the rotogram given by 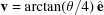, where 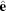 is the rotation axis, θ is the rotation angle and 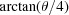 is a nonlinear scaling factor. Compared with the well-known axis-angle representation which maps a rotation to 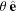 (*i.e.* the length of the vector encodes the rotation angle), this definition only slightly increases the computational burden and inherits its property of adjacent voxels corresponding to similar rotations. The nonlinear scaling of rotation angle to the length of the vector gives a more uniform distribution of points in the rotogram than the axis-angle representation. Our transform maps all possible rotations to a finite-size ball of radius 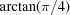 and is in some sense the opposite of the modified Rodrigues parameters, 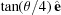.

The construction of 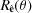 from 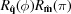 is achieved in a computationally inexpensive way by employing the composition law for finite rotations first derived by Olinde Rodrigues (Altmann, 1989 ▸; Pujol, 2013 ▸). This describes the consecutive operations of two general rotations 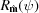 and 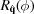 to give 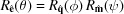 by solving 
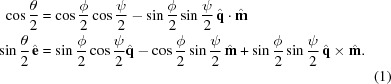
For our problem, the first rotation axis is the bisector 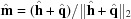, and 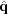 is the direction of the ULS. This choice of 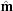 allows setting 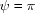 such that the equations simplify to 
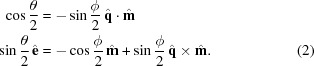
Setting the parameters 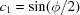, 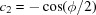, 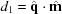 and 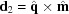 we obtain 
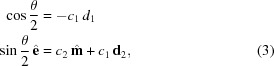
which can be solved as 
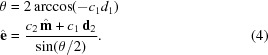
For machines where 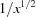 is implemented in hardware, replacing 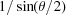 by 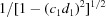 can lead to faster execution.

**Figure 5:**
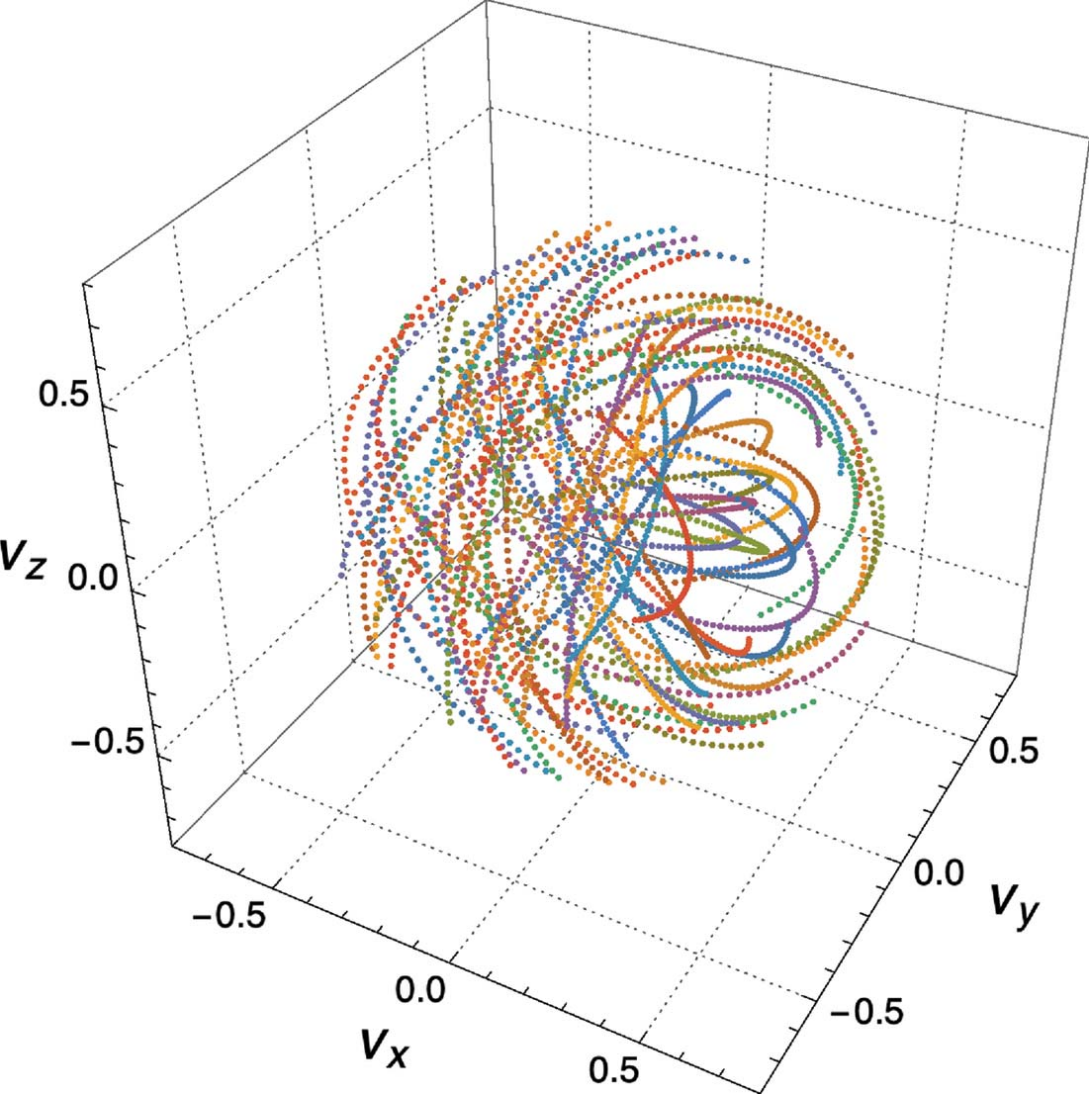
Plot of the curves 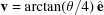 for a particular Bragg-point direction 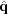 and a set of 56 candidate RLPs in a cubic lattice.

An example of a rotogram for a particular Bragg peak is shown in Fig. 5 ▸, for 56 candidate RLPs on a cubic lattice and a relative bandwidth of 6.5%. Each of the non-intersecting 56 colored curves is a plot of the vector 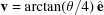 for a full rotation of the lattice. For a given point 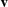 in the plot, the corresponding rotation angle of the lattice to bring the RLP onto the ULS defined by 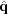 is 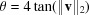, and the rotation axis is 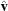. As seen from equation (2), each trajectory lies in the plane containing the orthogonal vectors 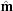 and 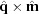, which is to say the plane normal to 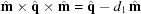. The trajectories form closed curves in the vector space over the range of ϕ from 0 to 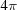, but we only require a single rotation of the lattice. To keep the rotogram volume as small as possible we choose the range 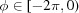. In the example of Fig. 5 ▸ the curves were sampled over that range at steps of 0.1 rad and while the curves are not necessarily uniformly sampled in the vector space, the choice of 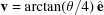 generates curves that more uniformly fill the space than the axis-angle representation 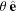 or any other construction that we tried.

In the implementation of *pinkIndexer*, rotograms are not calculated continuously as shown in Fig. 5 ▸ but computed on discrete sets of 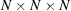 voxels that circumscribe the ball of radius 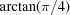. For each Bragg spot the voxel array is initialized with zeros and voxels are set to 1 that are intersected by the curve 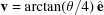 for each of the candidate RLPs, with a uniform sampling of ϕ that is chosen to ensure that the curve is contiguous across the voxels. This is accomplished by computing the parameters 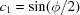 and 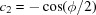 at those values once for the whole rotogram and using values of the parameters 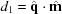 and 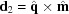 that need to be computed once per curve. To make the discretization of ϕ smoother, the flagged voxels are dilated by setting all of their 26 neighboring voxels to 1. This reduces the effective resolution of the rotogram, but increases the noise tolerance. The rotogram indicates all orientations of the crystal that predict the respective Bragg spot.

By adding each Bragg spot’s rotogram, a total rotogram is created where the value of each voxel gives the number of Bragg spots predicted by the corresponding orientation. The voxel with the maximum value thus indicates the most likely lattice orientation that provides the correct indexing solution. The task of indexing is thus reduced to finding the location of this maximum. Since the rotogram is discrete the determined indexing solution is approximate. A subsequent refinement is carried out to increase the precision of the indexing solution, in which the lattice basis is refined to minimize the mean Euclidean distance between the ULS and the respective closest RLP using a gradient descent approach. Only the RLPs close enough to a ULS are used for this refinement to improve noise tolerance.

### 2.4. Implementation details   

The algorithm has been implemented such that parameters have effects that are easy to understand. Besides experimental settings like detector distance and pixel geometry, beam parameters and crystal lattice parameters, *pinkIndexer* requires a relative tolerance, set by the parameter *tolerance*, to decide when a peak is correctly fitted. Additional parameters trade-off fitting performance against execution time. The parameter *consideredPeaksCount* specifies the number of found Bragg spots that are used to compute the initial indexing solution from the maximum of the rotogram. All Bragg spots are considered during refinement. The parameter *angleResolution* sets the resolution of the rotogram in terms of number of voxels *N* spanning 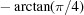 to 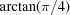. Choosing larger voxels (lower resolution) leads to a faster calculation but lower precision in the initial step of determining the orientation from the rotogram. The second step of refining the orientation is controlled by the parameter *refinementType*. Refinement can be performed by a gradient descent method, fitting all parameters of the lattice or keeping the cell parameters constant and just refining the orientation. All parameters take descriptive values wherever possible.

## 3. Evaluation of algorithm performance   

We evaluated the performance of the *pinkIndexer* algorithm on data from macromolecular crystal diffraction experiments utilizing three different types of radiation: monochromatic X-rays, pink X-rays and electrons. For the evaluation we used the *CrystFEL* (White *et al.*, 2012 ▸) software suite 0.8.0+50a3cb06 with modifications to include the *pinkIndexer* library and enable prediction for wide-bandwidth and electron beams.

### 3.1. Monochromatic X-ray beam crystallography   

The performance of *pinkIndexer* in treating monochromatic serial femtosecond X-ray diffraction data was compared with the indexers *MOSFLM* (Powell, 1999 ▸), *XDS* (Kabsch, 1993 ▸, 2010 ▸), *DirAx* (Duisenberg, 1992 ▸), *TakeTwo* (Ginn *et al.*, 2016 ▸), *FELIX* (Beyerlein *et al.*, 2017 ▸) and *XGANDALF* (Gevorkov *et al.*, 2019 ▸) using the *indexamajig* program from the *CrystFEL* (White *et al.*, 2012 ▸) software suite. For the test, all *CrystFEL* optimizations were turned off by using the options --no-retry --no-refine --no-multi --no-check-cell --no-check-peaks. Only one indexing solution per pattern was accepted. Indexing solutions that differed from the original indexing solution by less than 3° were counted as correct. The diffraction data set was retrieved from the CXIDB (Maia, 2012 ▸), entry 21, from SFX measurements of a G-protein-coupled receptor (the serotonin 5-HT_2B_ receptor bound to ergotamine) (Liu *et al.*, 2013 ▸).

**Figure 6:**
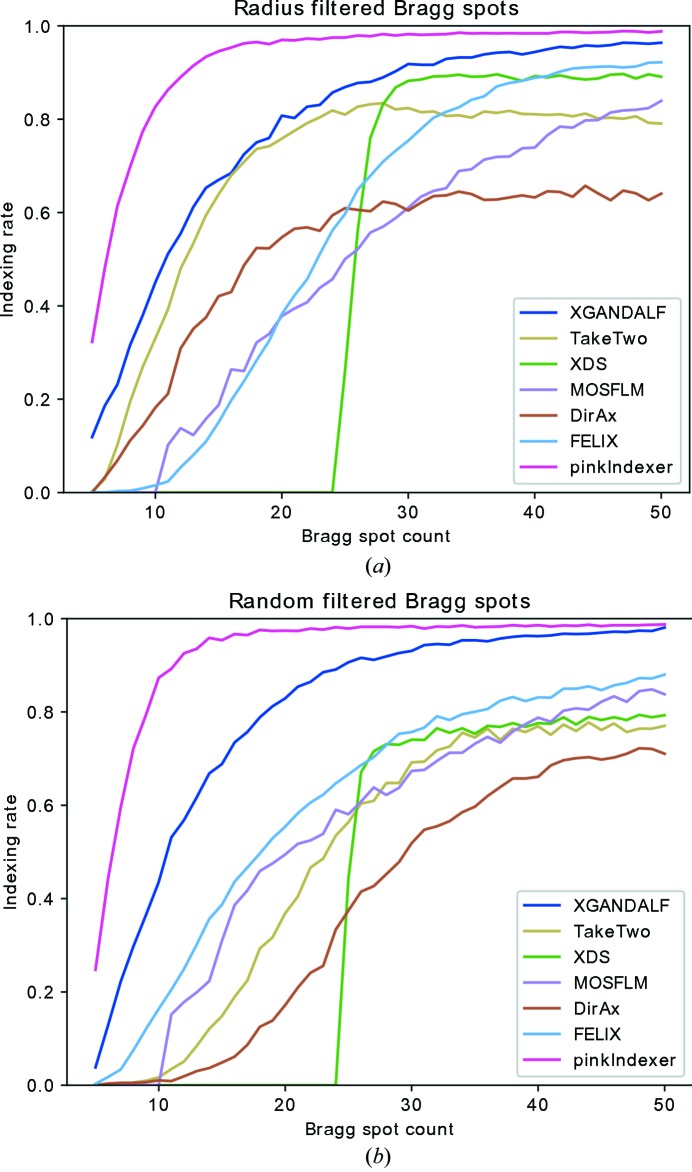
Comparison of *pinkIndexer* with other indexing algorithms, using the *CrystFEL* software suite. Each original experimental diffraction pattern had more than 50 Bragg spots and was indexable with *MOSFLM*. From every pattern, a number of Bragg peaks were selected to be used for indexing (indicated along the abscissa) either (*a*) by selecting those with the smallest scattering angles or (*b*) randomly. The indexing results were compared with the original *MOSLFM* indexing solution using all Bragg spots. Indexing solutions that differed from the original indexing solution by less than 3° were counted as correct.

Comparing algorithms using real data provides results that indicate their performance under real conditions. However, unlike when using simulated data, the true indexing solution is unknown. The indexing solutions can be tested for correctness by comparing the predicted Bragg spots with the found ones. This is a precise method when there are many Bragg spots, but when the number of found spots is small there can be several incorrect orientations of a crystal that predict the found spots well enough to pass the indexing test. Following a practice we introduced earlier to compare indexing algorithms (Gevorkov *et al.*, 2019 ▸) we created semi-simulated data sets with different numbers of Bragg spots by removing spots from patterns with large numbers of spots (which have reliable indexing solutions). As previously, we tested the indexers in two modes of Bragg-peak removal. In one mode the sets contained patterns with only five to 50 Bragg spots selected randomly from the patterns. In the other mode the sets of patterns contained five to 50 Bragg spots only at low resolution. The comparison is given in Fig. 6 ▸. All algorithms performed well when there were sufficient measured Bragg spots to determine the crystal lattice. With both cases of randomly distributed Bragg spots and low-resolution Bragg spots, the *pinkIndexer* algorithm outperformed all others over the whole range of Bragg-spot counts. The settings of *pinkIndexer* in these tests were chosen to favor precision over speed (*angleResolution* = ‘dense’). In all cases the lattice parameters were specified to the indexing algorithm. No additional tuning of the indexing algorithms was performed apart from an option that allows *FELIX* to index patterns with as few as five peaks.

The average times for the various algorithms to index monochromatic diffraction patterns are given in Table 1 ▸, computed by indexing a set of 1000 diffraction patterns chosen randomly from the same data set as used above. To ensure a fair comparison, all indexers were called from *CrystFEL* with the 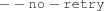 flag set. No attempt was made to index multiple crystals per pattern. The program was executed on a dual-socket Intel Xeon E5-2698 v4 CPU (2.20 MHz, 20 cores, 50 MB cache, 512 GB RAM). *pinkIndexer* was tested with settings to maximize the speed (‘fast mode’ in Table 1 ▸, *angleResolution* = ‘loose’) and with settings to maximize the yield (‘precise mode’, *angleResolution* = ‘dense’) which took about five times longer. Settings in-between are also possible. Even in the fast mode the algorithm takes considerably longer than other algorithms except for *TakeTwo*. The slower speed of *pinkIndexer* is because the algorithm is memory intense. Nevertheless, due to its high indexing success rate, *pink­Indexer* can be profitably used as a fallback option for monochromatic diffraction patterns that cannot be indexed by other indexers. This can be implemented in *CrystFEL* by placing *pinkIndexer* last in the list of indexers.

### 3.2. Pink-beam serial crystallography   

**Figure 7:**
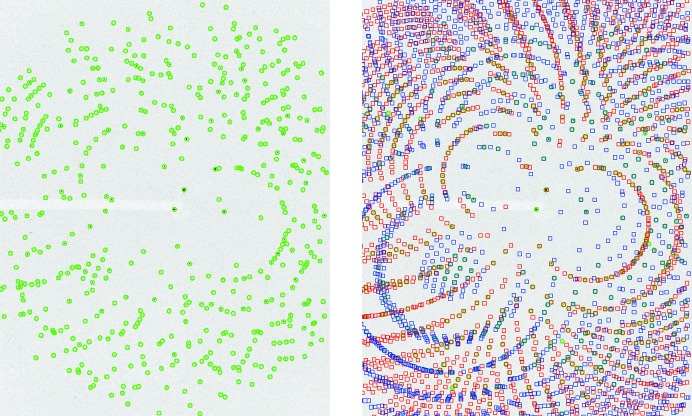
A snapshot pink-beam diffraction pattern of two proteinase K crystals measured coincidentally at the 14-ID-B (BioCARS) beamline at the APS. The pattern is overlaid with the detected peaks (left) and with detected as well as predicted peaks (right). Both crystals were indexed by *pinkIndexer* (red and blue). Note the agreement between prediction and measurement.

Diffraction patterns collected using pink-beam radiation (1% to 25% relative bandwidth) contain many observed peaks, which means that indexing solutions can easily be verified by comparing the predicted with the observed Bragg spots. To evaluate *pinkIndexer* using real pink-beam serial crystallography data we used the data set of proteinase K crystal diffraction from Meents *et al.* (2017 ▸) measured at the 14-ID-B (BioCARS) beamline at the Advanced Photon Source (APS), using the full polychromatic spectrum of an undulator harmonic. A representative diffraction pattern is depicted in Fig. 7 ▸. Although the FWHM of the incident X-ray beam spectrum was 5% of the mean photon energy, the tails of the spectrum extended up to 25% of the mean photon energy. The data set contained 999 patterns that had been classified as crystal diffraction ‘hits’ based on the detection of at least 35 Bragg spots in the original work of Meents *et al.* (2017 ▸). Of these, 667 patterns were successfully indexed by *pinkIndexer*, with 428 determined to contain a single lattice, 168 with two lattices, and 71 with three or more lattices. This gave a total of 1005 indexed lattices. A vast majority of the 332 patterns that could not be indexed appeared to be falsely identified as crystal diffraction patterns due to fitting peaks to noise. The *pinkIndexer* parameters used in this test are given in Appendix *A* [App appa].

The comparison of polychromatic indexing programs in an automated way for serial crystallography data sets is complicated by these programs not being able to be called from within the *CrystFEL* software suite. Indeed, the analysis of the pink-beam serial crystallography data carried out by Meents *et al.* (2017 ▸) could only be achieved in a semi-manual way using the software *Precognition*. This resulted in the indexing of 140 patterns of the 999 (Meents *et al.*, 2017 ▸), and only single-crystal diffraction patterns could be indexed. This comparison shows that, for serial crystallography, *pinkIndexer* provides an order of magnitude more indexable patterns than the current state-of-the-art software. Moreover, *pinkIndexer *can deal with multiple crystals per pattern and is fully automatic, thus making serial crystallography with a pink beam much easier. Fig. 7 ▸ shows two crystals contributing to the pattern which are both indexed correctly. We have also successfully used *pinkIndexer* for serial crystallography data from Tolstikova *et al.* (2019 ▸) measured with X-rays with 2.5% relative bandwidth produced using a multilayer monochromator.

### 3.3. Serial electron crystallography   

**Figure 8:**
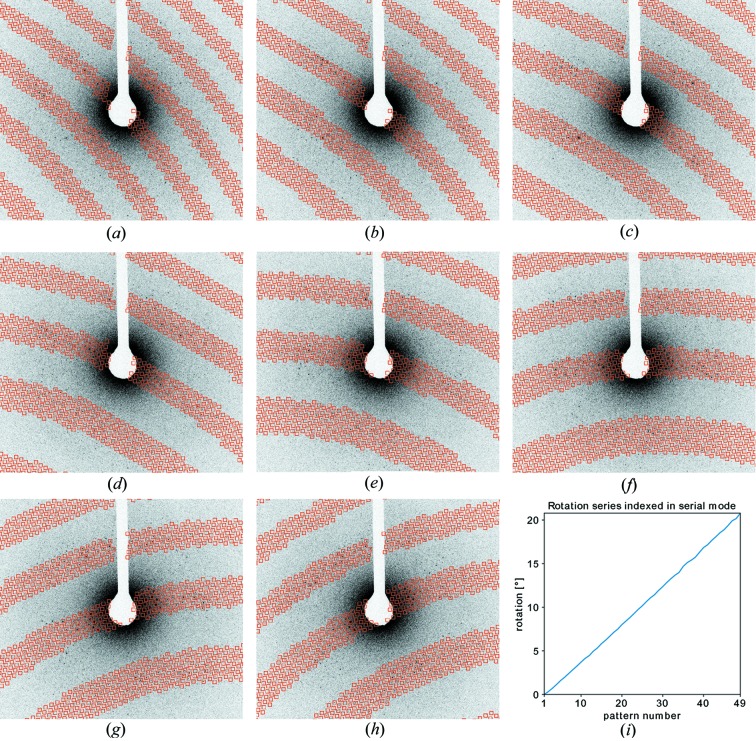
(*a*)–(*h*) Patterns 30–37 from the electron diffraction rotation series data of proteinase K in Cruz *et al.* (2017 ▸). The red squares mark the locations of the predicted Bragg spots after indexing. (*i*) Plot of the crystal rotation angle as derived from the indexing result of *pinkIndexer*.

Electron crystallography poses a challenge for conventional indexing algorithms due to flatness of the Ewald sphere caused by the short de Broglie wavelength of the electrons. To demonstrate the applicability of *pinkIndexer* to serial electron diffraction data, a rotation series data set from Cruz *et al.* (2017 ▸) was treated like serial data by indexing each pattern individually. The known rotation increment available for this data set was used to check the correctness of the indexing solutions. The results are displayed in Fig. 8 ▸. All patterns from the data set were indexed correctly, as can be seen from the linear increment of the determined rotation angle. The maximum deviation of the angle determined by indexing from a linear fit was 0.14°, which represents an upper bound to the indexing precision since goniometer errors may also contribute. This result opens up the possibility of serial electron crystallography using randomly oriented crystals exposed in individual data frames as performed in SFX measurements.

## 4. Conclusion   

The indexer presented in this paper has been developed for pink-beam serial crystallography using the full polychromatic spectrum of an undulator harmonic at a synchrotron radiation facility. Starting from known unit-cell parameters, the *pinkIndexer* algorithm works by mapping all possible rotations of candidate reciprocal-lattice points onto line segments in reciprocal space that correspond to Bragg peaks of unknown wavelength. By examining these mappings in a novel rotation space the most likely lattice orientation can be found. The main limitation of *pinkIndexer* is its slower speed compared with many other existing algorithms for monochromatic diffraction analysis and the requirement of knowing the cell parameters of the studied crystals. The benefit, however, is its higher success rate in indexing snapshot diffraction patterns than all other algorithms we tested.

Due to the generality of the approach to different wavelengths and spectral characteristics, the algorithm presented here has the ability to open up emerging and as-yet-unexplored avenues of serial crystallography. The higher X-ray flux of the polychromatic beam enables exposure times as short as those emitted from a single electron bunch in the storage ring, while the broad bandwidth leads to a high fraction of fully integrated Bragg peaks recorded in a snapshot pattern and a greater coverage of reciprocal space. These advantages have long been appreciated for time-resolved Laue diffraction experiments at synchrotron facilities (Moffat, 1997 ▸), macromolecular crystallography at neutron facilities (Blakeley *et al.*, 2008 ▸), and have motivated the generation of pulses with broader bandwidth at free-electron laser facilities (White *et al.*, 2013 ▸; Dejoie *et al.*, 2013 ▸). As demonstrated here, the *pinkIndexer* program overcomes difficulties previously encountered in automatically analyzing thousands of polychromatic diffraction patterns. Additionally, the generality of the algorithm makes it useful for indexing monochromatic serial crystallography. In this case we found that *pinkIndexer* demonstrates a superior success rate in indexing diffraction patterns, especially for the tricky case of a small number of detected Bragg spots. We also showed that the approach works well for indexing snapshot diffraction patterns recorded with very short wavelengths, which is usually the situation in electron diffraction. The method might additionally find application in neutron diffraction and could be slightly modified to treat the case of convergent-beam diffraction.

## 5. Code availability   

*pinkIndexer* is implemented in C++ and released as an open-source library under the LGPLv3 licence. This library can be compiled independently or together with the program suite *CrystFEL* (White *et al.*, 2012 ▸). The full processing pipeline, including indexing, high-precision prediction and integration, will be realized soon as a part of *CrystFEL*. The source code can be downloaded at https://stash.desy.de/users/gevorkov/repos/pinkindexer/browse.

## Figures and Tables

**Table 1:** Indexing results and execution times for monochromatic indexers and *pinkIndexer*

Indexer name	Indexed patterns	Total execution time [(mm:ss)/per pattern (ms)]
*MOSFLM*	452	00:17/17
*XDS*	400	00:22/22
*DirAx*	394	00:12/12
*TakeTwo*	545	11:02/662
*FELIX*	656	01:05/65
*XGANDALF*	724	00:19/19
*pinkIndexer* fast mode	757	04:15/255
*pinkIndexer* precise mode	816	22:16/1336

## Appendix A *pinkIndexer* settings

The *pinkIndexer* settings for Section 3.2 were chosen as follows (format as used by *CrystFEL*):

photon_energy = 11000,

photon_energy_bandwidth = 0.25,

--pinkIndexer-considered-peaks-count=4,

--pinkIndexer-angle-resolution=3,

--pinkIndexer-refinement-type=4,

--pinkIndexer-tolerance=0.03.

Unit cell:

lattice_type = tetragonal,

unique_axis = c,

centering = P,

a = 69.10 A,

b = 69.10 A,

c = 106.60 A,

al = 90.00 deg,

be = 90.00 deg,

ga = 90.00 deg.

*pinkIndexer* settings for Section 3.1 were chosen as follows (format as used by *CrystFEL*):

--pinkIndexer-tolerance=0.13,

--pinkIndexer-angle-resolution=3.

The *XGANDALF* settings for Section 3 were chosen as follows (format as used by *CrystFEL*):

--xgandalf-sampling-pitch=2,

--xgandalf-grad-desc-iterations=2,

--xgandalf-tolerance=0.02.

The *FELIX* settings for Section 3 were chosen as follows (format as used by *CrystFEL*):

--felix-max-visits=5.
